# Ectopic Noggin in a Population of Nfatc1 Lineage Endocardial Progenitors Induces Embryonic Lethality

**DOI:** 10.3390/jcdd1030214

**Published:** 2014-11-20

**Authors:** Paige Snider, Olga Simmons, Jian Wang, Chinh Q. Hoang, Simon J. Conway

**Affiliations:** Developmental Biology and Neonatal Medicine Program, HB Wells Center for Pediatric Research, Indiana University School of Medicine, Indianapolis, IN 46202, USA

**Keywords:** mouse embryo, transgenic overexpression, Noggin, Nfatc1, cardiac endocardial cushions, endocardium-cardiomyocyte cross-talk

## Abstract

The initial heart is composed of a myocardial tube lined by endocardial cells. The TGFβ superfamily is known to play an important role, as BMPs from the myocardium signal to the overlying endocardium to create an environment for EMT. Subsequently, BMP and TGFβ signaling pathways synergize to form primitive valves and regulate myocardial growth. In this study, we investigated the requirement of BMP activity by transgenic over-expression of extracellular BMP antagonist Noggin. Using *Nfatc1^Cre^* to drive lineage-restricted Noggin within the endocardium, we show that ectopic *Noggin* arrests cardiac development in E10.5-11 embryos, resulting in small hearts which beat poorly and die by E12.5. This is coupled with hypoplastic endocardial cushions, reduced trabeculation and fewer mature contractile fibrils in mutant hearts. Moreover, *Nfatc1^Cre^*-mediated *diphtheria toxin fragment-A* expression in the endocardium resulted in genetic ablation and a more severe phenotype with lethality at E11 and abnormal linear hearts. Molecular analysis demonstrated that endocardial Noggin resulted in a specific alteration of TGFβ/BMP-mediated signal transduction, in that, both Endoglin and ALK1 were downregulated in mutant endocardium. Combined, these results demonstrate the cell-autonomous requirement of the endocardial lineage and function of unaltered BMP levels in facilitating endothelium-cardiomyocyte cross-talk and promoting endocardial cushion formation.

## 1. Introduction

Following differentiation from multipotent cardiac field progenitors, the endocardial cells give rise to an internal epithelial layer adjacent to the myocardium of the initially linear embryonic heart [1–3]. They form an endocardial tube by vasculogenesis and subsequently become the endocardium of the heart [4]. As development proceeds, endothelium-cardiomyocyte cross-talk helps to form specialized structures of the heart such as heart valves and myocardial trabeculae [5].

Concomitant with looping to enable formation of a four-chambered heart, trabeculation initiates and primitive cardiomyocytes form highly organized muscular ridges that are lined by layers of invaginated endocardial cells [6,7]. Several studies have established that the endocardial lineage plays an important role in spatial-temporal control of myocardial trabecular growth via endocardial Notch1-mediated Bmp10/Neuregulin-1/ErbB signaling in adjacent cardiomyocytes [8–11], and that endocardial Fkbp1a is required for modulation of Notch1 activity during ventricular wall formation [12]. Conversely, VEGF and Angiopoietin signal from the myocardium to the endocardium to regulate trabeculation [13,14]. In parallel, valve formation originates from a subpopulation of endocardial cells lining the myocardium that undergo endothelial-mesenchymal transition (EMT), proliferate and migrate into the extracellular matrix/cardiac jelly to form bilateral cardiac cushions in both the atrioventricular canal and the outflow tract [5,15,16]. Appropriate placement of the cushions is thought to be the development of the primary heart tube as a segmented organ, as well as the subsequent looping of the heart [17]. Significantly, Bmp2 [18,19], Bmp4/Bmp6 [20] and Msx1/Msx2 [21] all exhibit restricted expression within the AV canal and outflow tract myocardium and are thought to be inducers of endocardial cushion. Similarly, in addition to Notch [11] and its ligands Jag1 and Jag2 [22], Type I BMP receptors, ALK2 and ALK3 and TGFβ superfamily co-factor Smad4 [23] are present in the adjacent endocardium [19,24] and are thought to initiate EMT [25]. Subsequently, both BMP and TGFβ signaling synergize with Notch to promote the transition of endothelia to mesenchyme and the mesenchymal cell invasiveness [26,27].

BMPs have diverse roles in cardiomyocyte formation and maturation, valve development, trabeculation and embryonic cardiac morphogenesis. Moreover, numerous BMP ligands and receptors are expressed at high levels in the developing mouse heart and both systemic and conditional knockout mouse approaches have demonstrated that the majority of individual components play critical roles in early cardiac development [26]. However, the detailed molecular mechanisms regulated by BMP remain poorly understood due to the large number of family members with overlapping functions, parallel pathways, genetic redundancy and functional compensation. In order to begin to understand the role that blanket BMP signaling suppression may play in congenital heart defect pathogenesis and the spatiotemporal requirement of BMP function within the embryonic endocardium, we used the endocardially-restricted *Nfatc1^Cre^* knockin line to induce tissue specific expression of the BMP extracellular antagonist Noggin. Importantly, the endocardium comprises a unique endothelial cell population that expresses Nfatc1 during development, whereas vascular endothelial cells do not express Nfatc1 [28–30]. Additionally, although endogenous Noggin is transiently expressed in the heart-forming region during gastrulation and is thought to act at the level of induction of mesendoderm to establish conditions conducive to cardiogenesis [31] and systemic loss of Noggin results in mutant mouse embryos with a thicker myocardium and larger endocardial cushions [32], endogenous Noggin is not present in either the E9.5 and older myocardium nor endocardium [19]. Indeed, exogenous Noggin blocks AVC explant EMT in culture [18]. Herein, we show that ectopic Noggin expression within the endocardium results in embryonic lethality, undersized bradycardic hearts with immature cardiomyocyte contractile apparatus, hypoplastic endocardial cushions and both BMP and TGFβ downstream effector altered expression profiles. Moreover, a similar phenotype but earlier time of lethality was observed when the endocardium was genetically ablated. Taken together, these results indicate that a distinct level of BMP activity is necessary for endocardial-cardiomyocyte cross-talk, and that suppression of BMP signaling results in both heart valve and myocardial trabeculae defects within the developing mouse heart.

## 2. Materials and Methods

Genetically modified mouse models: *pMes-Noggin* floxed conditional overexpression mouse model [33] was crossed with *Nfatc1^Cre^* knockin mice [3] to generate mutant embryos expressing *Noggin* within the endocardium and cushion mesenchyme derived from the endocardial lineage. *pMes-Noggin/Nfatc1^Cre^* mice (hereafter called *Nog^End^*) were placed on *ROSA26r* (*R26r*) indicator background [34] for lineage mapping and to assess lineage-restricted Cre-mediated induction of *Noggin* expression. Yolk sac or tail tissue genomic DNA was genotyped, using two sets of primers: for *Cre* (5’-AATAAGCCTGCCGTGGTCACTGG; 3’-AACCCTGGACGCCTGGGACAC for detection of wildtype and 5’-GAAGCAACTCATCGATTGATTTACG; 3’-AACCCTGGACGCCTGGGACAC for detection of mutant) and *Noggin* (5’-CCCCCTGAACCTGAAACATA; 3’-GGCGGATGTGTA GATAGTGCT). *Nfatc1^Cre^* knockin mice were crossed to *ROSA26^eGFP DTA^* mice (*R26r^DTA^*; [35]) to genetically ablate the *Nfatc1^Cre^* lineage *in utero* and genotyped as previously described [36]. Animal procedures and experimental conditions were refined to minimize harm to animals and performed with the approval of the Institutional Animal Care and Use Committee of Indiana University School of Medicine.

Measuring heartbeat: Individual whole E11 embryos (with deciduae and embryonic blood vessels left attached) were dissected from the mother in 37 °C DMEM medium supplemented with 5% fetal calf serum (Gibco-BRL), placed in a closed 12 well culture tray and allowed to recover for 10 min in incubator (37 °C, 5% CO_2_), as previously described [37]. Each embryo was transilluminated and the heartbeat digitally recorded using an AxioCam MRc camera and dissecting scope (Zeiss) for 5 min and then PCR genotyped retrospectively. Heart rates were determined via calculating cardiac contractions/min in 7 control and 6 *pMes-Noggin/Nfatc1^Cre^* mutants (*n* = 4 litters).

Immunohistochemistry, histology and X-Gal staining: Isolation of tissues, fixation, processing, and whole mount staining for β-galactosidase and hematoxylin/eosin counterstaining was performed as described [36,38,39]. Subsequently, fixed embryos were sectioned at 6 μm thickness. ABC kit (Vectorstain) with DAB and hydrogen peroxide as chromogens was used for signal detection as described [40]. The following primary antibodies were used: phospho-Smad1/5/8 (1:40,000, Cell Signaling), α-Smooth muscle actin (1:5000, Sigma, St. Louis, MO, USA), PECAM-1 (1:200, BD Biosciences Pharmingen, San Jose, CA, USA) and Periostin (1:10,000) as described [41]. For each assay, whole embryos and/or serial sections were examined for at least three individual embryos of each genotype at each stage of development. Collected from nine continuous sections of three individual samples of wildtype controls and mutants, respectively, data were subjected to Student's t-test to determine the significance of differences. Wildtype littermates were always used as age-matched control samples (*p* values were assigned, with <0.05 being significant).

Analysis of proliferation and apoptosis: Cell proliferation was examined via phospho-histone H3 (1:500, Millipore) immunohistochemistry. Transverse serial sections of 6 μm paraffin-embedded embryos were stained with an antibody against pHH3 to identify mitotic cells and with hematoxylin to identify nuclei. DAB-positive cells were counted within both the atria and ventricles of 12 non-overlapping fields from at least 6 slides per embryo, and at least 3 embryos per genotype. The outcome of pHH3 labeling was presented as percentage of labeled cells among total nuclei in the fixed hearts. Cell apoptosis was evaluated using TdT-FragEL™ DNA Fragmentation Kit (Calbiochem) following manufactures protocol. Apoptosis was determined via counting DAB-positive cells per 10,000 ventricular cells counted from at least 6 slides per embryo, and at least 3 embryos per genotype.

Confocal immunofluorescence: Embryos (*n* = 4 for each age group and genotype) were collected in ice cold PBS, fixed in 4% paraformaldehyde, and placed in sequentially higher sucrose solutions. Specimens were embedded in Tissue-Tek OCT, frozen at −80 °C and sectioned at 5 μm. Immunostaining was performed using Vector Labs MOM fluorescent kit (FMK-2201) following the manufacturer's protocol. Rhodamine phalloidin (1:100, Molecular Probes) and α-Actinin (1:400, Sigma) were used. Stained sections were photographed using Zeiss Axioskop-2 plus microscope and AxioVision Rel.4.8 software.

*In situ* hybridization and western blotting: Both radioactive and non-radioactive *in situ* hybridization analysis of *Endoglin, Msx2* and *Tgfβ1* expression were performed as described [42] using anti-sense S^35^-labeled and DIG-labeled (DIG RNA labeling mix, Roche) cRNA probes. *Endoglin* and *Msx2* probes were provided by Helen Arthur [43] and Robert Maxson [21], and *Tgfβ1* probe was described previously [44]. For all of these probes, serial sections were examined using at least three individual E10.5 embryos of each genotype. Western blotting was performed as described [45]. Proteins from E10.5 isolated control and *Nog^End^* hearts were probed with phospho-Smad1/5/8 (1:1,000, Cell Signaling), phospho-Smad3 (1:1,000, Epitomics), phospho-Smad2 (1:1,000, Cell Signaling), and loading control Tubulin (1:10,000, Sigma). Signal was detected via ECL-plus (Amersham) and densitometric quantification of Western data (*n* = 4 pooled hearts per genotype) was analyzed using the AlphaVIEW SA program. qPCR analysis: To verify *Noggin* induction in *Nog^End^* mutants, we used qPCR on cDNA synthesized from pooled (*n* = 4 hearts of *Nog^End^* and wildtype) E9.5 isolated hearts using a Superscript-II kit (Invitrogen). cDNA was amplified within the linear range using *Noggin* primers and normalized with *GAPDH* as described [40]. For expression profiling, mRNA from E10.5 isolated hearts (3 hearts per genotype) was isolated using RNAEasy (QIAGEN) kit and reverse transcribed using SuperScript II Reverse Transcriptase. Two samples/genotype were used in the Mouse TGFβ/Bmp Signaling Pathway RT^2^ Profiler PCR Array (Qiagen) and qPCR was done in technical triplicate for each sample. qPCR reaction was carried out using SyberGreenER (Invitrogen). All qPCR results are representative of three separate experiments (*n* = 3 plates/genotype) and ΔΔC_t_ based fold-changes were calculated using GAPDH as a standard.

Statistical analysis: Statistical analysis was performed with Prism software (Graphpad Software, San Diego, CA, USA). Comparisons between experimental groups were made using unpaired student's *t* test. A *p*-value < 0.05 was considered statistically significant. Densitometry analysis was examined for statistical significance using Student's t-test. For statistical analysis of quantitative PCR data, pooled cDNA from wildtype and *Nog^End^* hearts (*n* = 3 pooled E10.5 samples for each genotype) were analyzed in triplicate for each transcript. Error bars indicate standard error of the mean (SEM).

## 3. Results

### 3.1. Ectopic Noggin in the Nfatc1 Endocardial Lineage is Embryonic Lethal

**Figure 1:**
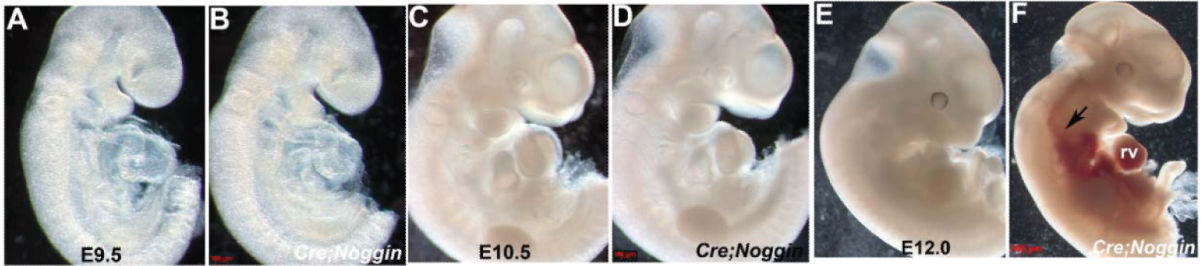

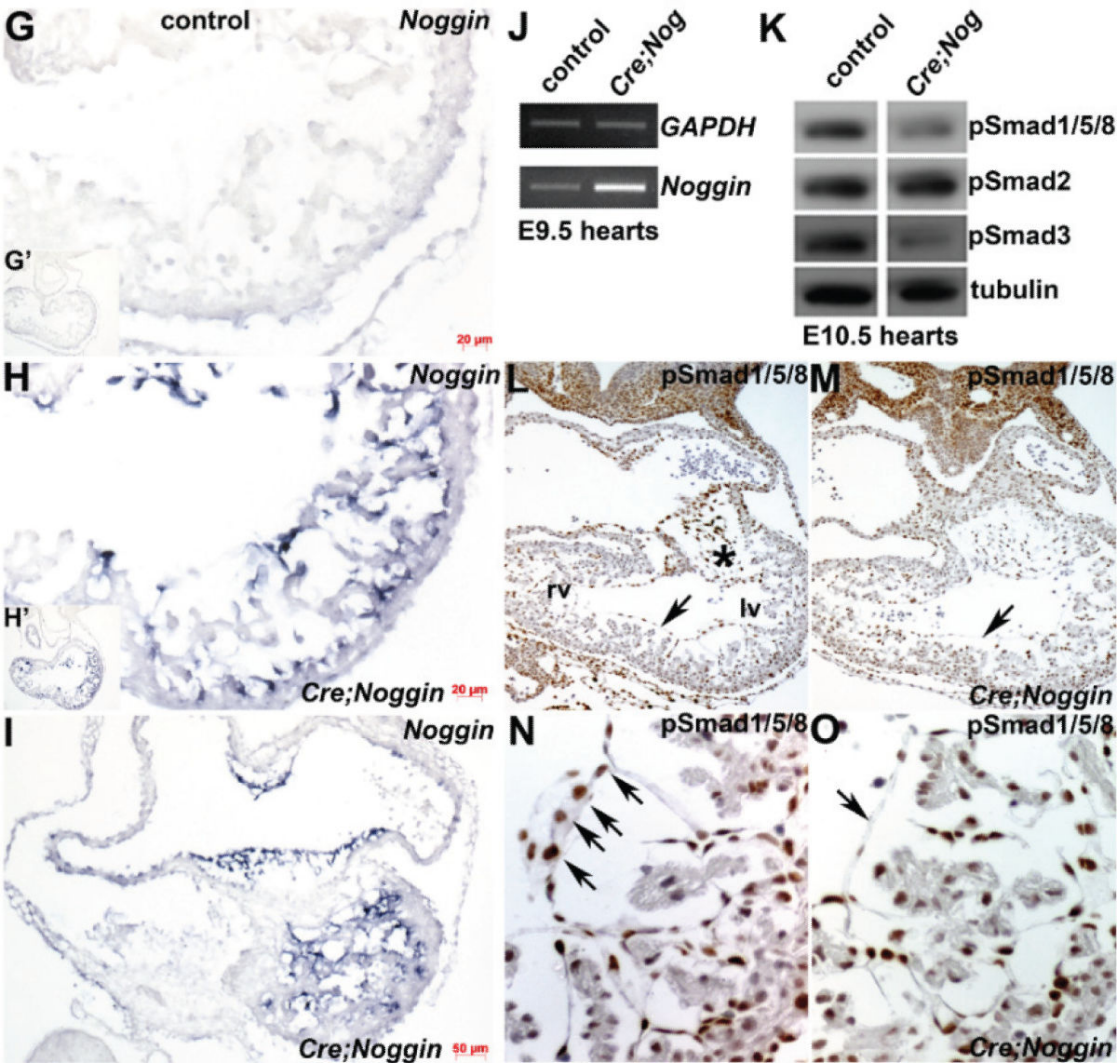
Ectopic *Noggin* in the Nfatc1 endocardial lineage is embryonic lethal. (**A**–**F)**: Resultant phenotype of *Nog^End^* mutant embryos (**B**,**D**,**F**) compared to wildtype littermate controls (**A**,**C**,**E**) at E9.5, 10.5 and 12. Wholemount right sided views show that E9.5 and 10.5 *Nog^End^* embryos are grossly unaffected but that E12 *Nog^End^* mutants are runted and exhibit pooling of blood in both blood vessels (indicated via arrow in **F**) and heart itself when compared to wildtype control littermates; (**G**–**I**): Non-radioactive *in situ* hybridization analysis using an anti-*Noggin* DIG-labelled probe confirms absent endogenous mRNA expression in control E10 heart (**G**) but robust endocardially-restricted transgenic *Noggin* (blue signal) in *Nog^End^* mutant heart within cells lining the atria, atrioventricular cushions and ventricle. **G’** and **H’** inserts represent low power images of whole heart sections; (**J**): Semi-quantitative RT-PCR measurement of *Noggin* levels in control and mutant E9.5 pooled hearts (*n* = 4 of each genotype) confirmed elevated *Noggin* expression levels (×6.4) in the mutant hearts (data shown from 34 PCR cycles). Note, *GAPDH* loading control (upper panel J) is similar in both samples (data shown from 20 PCR cycles); (**K**): Western analysis of TGFβ superfamily downstream effector Smad signaling in pooled E10.5 hearts (*n* = 4 of each genotype) revealed *Nog^End^* mutant isolated hearts exhibit reduced pSmad1/5/8 levels (only ~35% of wildtype levels) when normalized to Tubulin levels, indicative of suppressed downstream BMP ligand signaling. Additionally, pSmad3 is similarly reduced in mutant hearts (only ~40% of wildtype levels), but pSmad2 levels remain unchanged. This suggests that downstream TGFβ ligand signaling is also partially suppressed; (**L**–**O**): Immunohistochemical analysis of pSmad1/5/8 in E10.5 control (**L**,**N**) and mutant (**M**,**O**) hearts demonstrates that expression is unaffected in cardiomyocytes but that pSmad1/5/8 expression (brown nuclear staining) is reduced in *Nog^End^* mutant cushions compared to controls (* in (**L**)) and that pSmad1/5/8 expression is diminished within mutant endocardial cells (arrow in (**O**)) compared to wildtype controls (arrows in (**N**)). Scale bars: (**G**,**H**) = 20 μm; I = 50 μm. Abbreviations: lv, left ventricle; rv, right ventricle.

To assess the effects of BMP signaling suppression upon endocardial cell morphogenesis and function, we crossed *Nfatc1^Cre^* knockin mice [3] to *pMes-Noggin* transgenic mice [33] that have the mouse *Noggin* coding sequence cloned in front of the *IRES-Egfp* sequence under the control of the chick β-*actin* promoter, with a floxed STOP cassette inserted between the β-*Actin* promoter and the *Noggin* cDNA. As Noggin is a known secreted extracellular BMP antagonist [46,47] and *Nfatc1^Cre^* initiates Cre expression around E9 in the endocardium and is heart-restricted [3], thus over-expressing *Noggin* within the *Nfatc1^Cre^* lineage will suppress BMP signaling throughout early heart growth and endocardial development. These matings did not result in the birth of binary *pMes-Noggin/Nfatc1^Cre^* transgenic mutant mice (hereafter referred to as *Nog^End^* mutants), although other genotypes were present at expected Mendelian ratios and were normal (*n* = 7 litters). Genotyping of embryos during gestation allowed recovery of mutant embryos at the expected ratios between E9 and 12.5; however, no viable *Nog^End^* embryos were observed past E13 (*n* = 7 litters). At E9.5 and 10.5 *Nog^End^* embryos were comparable in size and appearance as wildtype littermates (Figure 1A–D). However, by E11 *Nog^End^* embryos appeared pale due to a decrease in circulating red blood cells within the mutant yolk sac, likely caused by a detectable decrease in heartbeat (*n* = 4 litters; controls= 273 ± 20 beats/min; *Nog^End^*= 189 ± 34). Moreover, by E12 *Nog^End^* mutants exhibit pooling of blood and undersized hearts when compared to wildtype littermates (Figure 1E,F). Additionally, the E12 mutant embryos were runted, the pharyngeal arches were hypoplastic and none survived until E13 (*n* = 7 litters).

### 3.2. Molecular Characterization of Nog Phenotype

As endogenous *Noggin* is not thought to be expressed in the E9.5 and older heart, and it is unclear whether the early embryonic endocardium expresses *Noggin* itself [19], we used non-radioactive *in situ* hybridization analysis to evaluate the spatiotemporal extent of endogenous and ectopic *Noggin* mRNA upregulation following *loxP/Cre* recombination in binary transgenic *Nog^End^* mutants. As previously described [19], we confirmed that endogenous *Noggin* is expressed in the E8 control notochord and transiently within the E8.5 heart (not shown), but no endogenous *Noggin* is observed in control E10 hearts (Figure 1G). In contrast, robust ectopic *Noggin* expression is present in the E10 *Nog^End^* heart, specifically within the endocardium lining the atria and ventricles as well as the endocardially-derived cushion mesenchymal cells of the atrioventricular canal (Figure 1H,I) but is absent from the adjacent cardiomyocyte lineage and proepicardium/epicardium. To determine the level of transgenic *Noggin* induction, we used semi-quantitative RT-PCR measurement of pooled hearts (*n* = 4 hearts of *Nog^End^* and wildtype). Significantly, at both 30 and 34 cycles, *Noggin* was unregulated ×6.4 fold in isolated *Nog^End^* mutant E9.5 hearts (Figure 1J). To define when and to what extent TGFβ superfamily signaling is suppressed via ectopic *Noggin* within the *Nog^End^* endocardial lineage, we used Western blotting to determine resultant SMAD intracellular signal transducer expression levels (as there is no Noggin antibody). As TGFβ superfamily signaling from the cell membrane to the nucleus occurs via SMAD proteins [48], as expected, phospho-SMAD1/5/8 expression (a downstream effector of BMP ligand signaling) was suppressed ~65% in isolated E10.5 *Nog^End^* hearts when compared to littermate controls (Figure 1K). Significantly, phospho-SMAD3 expression (a downstream effector of TGFβ ligand signaling) was also suppressed ~60% in isolated E10.5 *Nog^End^* hearts, but phospho-SMAD2 expression (another downstream effector of TGFβ ligand signaling) remained unchanged (Figure 1K). Immunohistochemistry confirmed that phospho-SMAD1/5/8 expression was indeed suppressed in the *Nog^End^* endocardial lineage and endocardially-derived cushions (Figure 1M,O), but not within the adjacent myocardium. pSMAD1/5/8 DAB-positive cells were counted within endocardium, trabeculated myocardium and AV/OT myocardium of three E10.5 control and mutant hearts. Significantly, per 10,000 cells counted, *Nog^End^* endocardium exhibited 42% less nuclear staining (2809 ± 14 stained mutant cells *vs.* 4895 ± 5 stained control cells; *p* 0.05). The E10.5 *Nog^End^* trabeculated myocardium and AV/OT myocardium were largely unaffected (mutant trabeculated myocardium exhibited 4511 ± 29 positive cells and the mutant AV/OT myocardium exhibited 5133 ± 22 positive cells per 10,000 total cells; whilst the control trabeculated myocardium exhibited 4604 ± 11 positive cells and the control mutant AV/OT myocardium exhibited 5002 ± 17 positive cells per 10,000 total cells; *p* 0.05). Jointly, these data show that *Nfatc1^Cre^*-mediated *Noggin* overexpression suppresses SMAD-dependent BMP and TGFβ signaling within only *Cre*-positive endocardium.

### 3.3. R26r Reporter Analysis of Noggin Overexpressing Lineages

**Figure 2:**
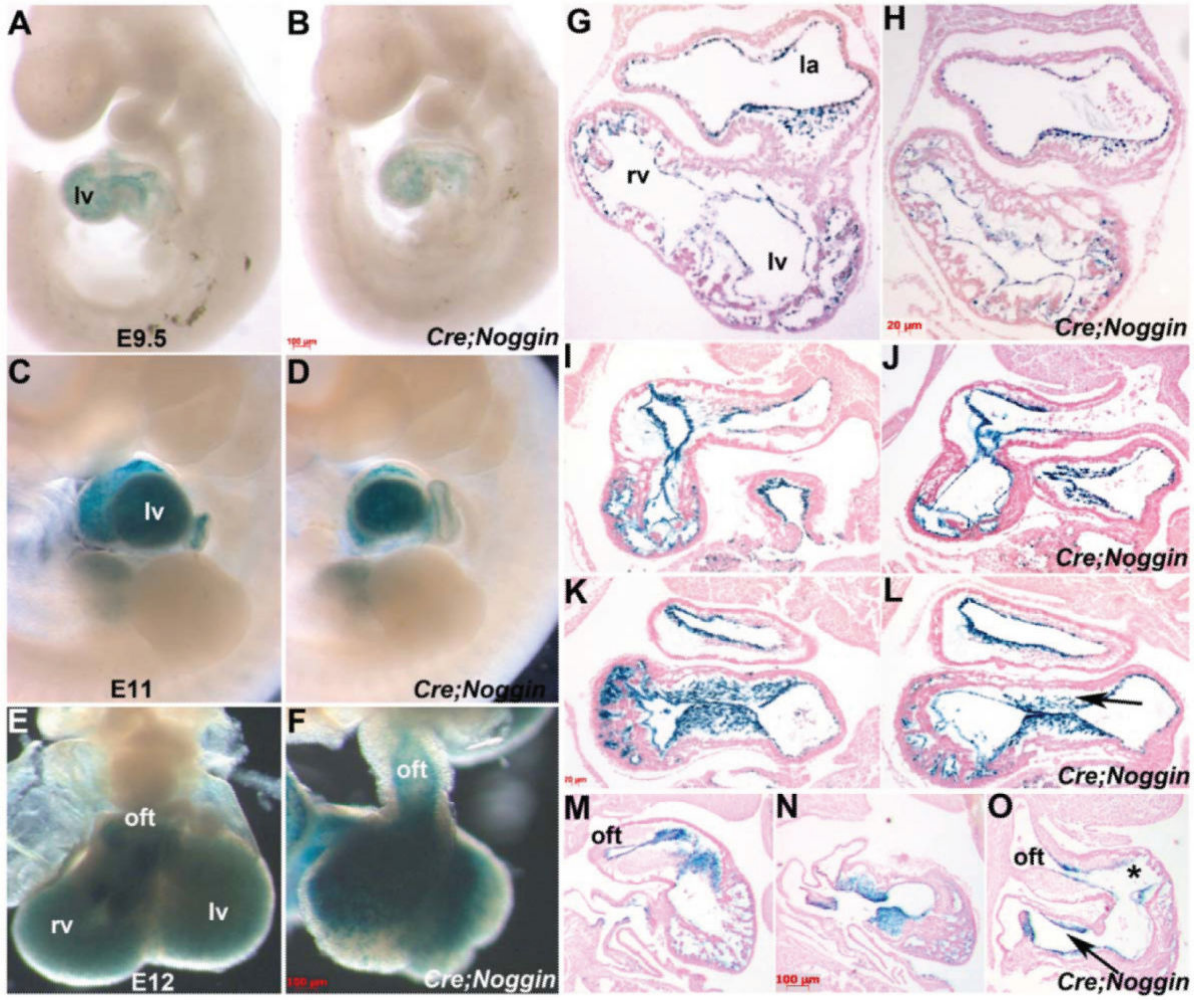
*R26r* reporter analysis of Noggin overexpressing lineages. (**A**–**D**): Left sided views of wholemount X-gal staining of control *Nfatc1^Cre^/R26r* (**A**,**C**) and mutant *pMes-Noggin/Nfatc1^Cre^/R26r* (**B**,**D**) embryos at E9.5 (**A**,**B**) and E11 (**B**,**D**) developmental stages, showing that Cre recombinase-mediated *lacZ* expression (blue) is restricted to the heart. Note *lacZ* staining reveals no obvious differences between control and *Nog^End^* mutants hearts, although the E11 *Nog^End^* mutant's heart is smaller; (**E**,**F**): Isolated control and *Nog^End^* E12 hearts viewed frontally, reveals that *Nfatc1^Cre^/R26r*–mediated *lacZ* reporter expression is still present in mutant heart (**F**) but that it is highly dysmorphic, misshapen and undersized; (**G**–**O**): Representative matched views of control E10 (**G**), E10.5 (**I**,**K**), E11 (**M**,**N**) and *Nog^End^* mutant E10 (**H**), E10.5 (**J**,**L**), E11 (**O**) serial sections X-gal stained and counterstained with eosin. Transverse sections of E10 control (**G**) and *Nog^End^* hearts (**H**) showed that *lacZ* expression was similarly localized in the endocardium and endocardial-derived cushion mesenchymal cells at the atrioventricular canals. E10.5 *lacZ* stained sagittal heart sections revealed that *Nog^End^* mutant atrioventricular cushion is reduced in cellularity (arrow in (**L**)) when compared to control, littermate (**K**); Additionally, the *Nog^End^* endocardium is detached from the underlying mutant trabeculae (**J**,**L**); Transverse sections of the X-gal stained E11 control (**M**,**N**) and mutant (**O**) hearts illustrates the hypoplastic endocardially-derived OFT (indicated via *) and atrioventricular cushions (arrow in (**O)**), the reduced trabeculae and detached endocardial lining; Note however, that *Nog^End^* neural-crest-derived OFT cushions are grossly normal in cellularity (**O**); Scale bars: (**A**–**F**) = 100 μm; (**G**–**L**) = 20 μm; (**M**–**O**) = 100 μm. Abbreviations: la, left atria; lv, left ventricle; oft, outflow tract; rv, right ventricle.

As gross morphology established that overexpression of *Noggin* from E9 onwards within only the endocardium results in fully penetrant *in utero* lethality by E13; we placed *pMes-Noggin/Nfatc1^Cre^* mice onto the *ROSA26r* reporter background [34] to follow the fate of the *Noggin* overexpressing lineage and determine whether TGFβ superfamily suppression resulted in abnormalities in lineage specification as well as morphogenesis. Significantly, as expected, wholemount *lacZ* staining is heart-restricted and still present in E9.5 and E11 control and *Nog^End^* mutant embryos (Figure 2A–D). Moreover, *lacZ* reporter is still present within E12 *Nog^End^* hearts (Figure 2F) and persist until subsequent ~E13 embryo lethality, despite their dysmorphic appearance and reduced size, indicating that the endocardium survives ectopic *Noggin* induction. Consistent with the gross defects observed, histology confirmed that E10 and E10.5 *Nog^End^* endocadium is intact and continuously lines the trabeculae and interior of the heart (Figure 2G–O). Moreover, the E10 *Nog^End^* heart structure and appearance is similar to control littermates, despite ectopic *Noggin* being present for at least ~6–12 h (Figure 2H). However, the E10.5 and E11 *Nog^End^* atrioventricular and outflow tract cushions are reduced in cellularity and the endocardium is detached from the underlying mutant trabeculae (Figure 2J,L,O). Additionally, trabeculation of the E10.5 and 11 *Nog^End^* mutant heart ventricles is blunted, when compared to control littermates (Figure 2J,O). The trabeculation phase of cardiac development is initiated at the end of cardiac looping (E9.0 to E9.5 in mouse) and is defined as the growth of primitive cardiomyocytes to form the highly organized muscular ridges that are lined by layers of invaginated endocardial cells [6,12]. Normally, newly formed trabeculae gradually contribute to the formation of the papillary muscles, the interventricular septum and compact at their base adjacent to the outer myocardium, adding substantially to ventricular wall thickness [7]. However, the *Nog^End^* mutant trabeculae fail to continue their expansion and outgrowth, and the mutant endocardium is detached (Figure 2J,O).

### 3.4. Immunohistochemical Analysis of Nog^End^ Mutant Growth and Development

**Figure 3:**
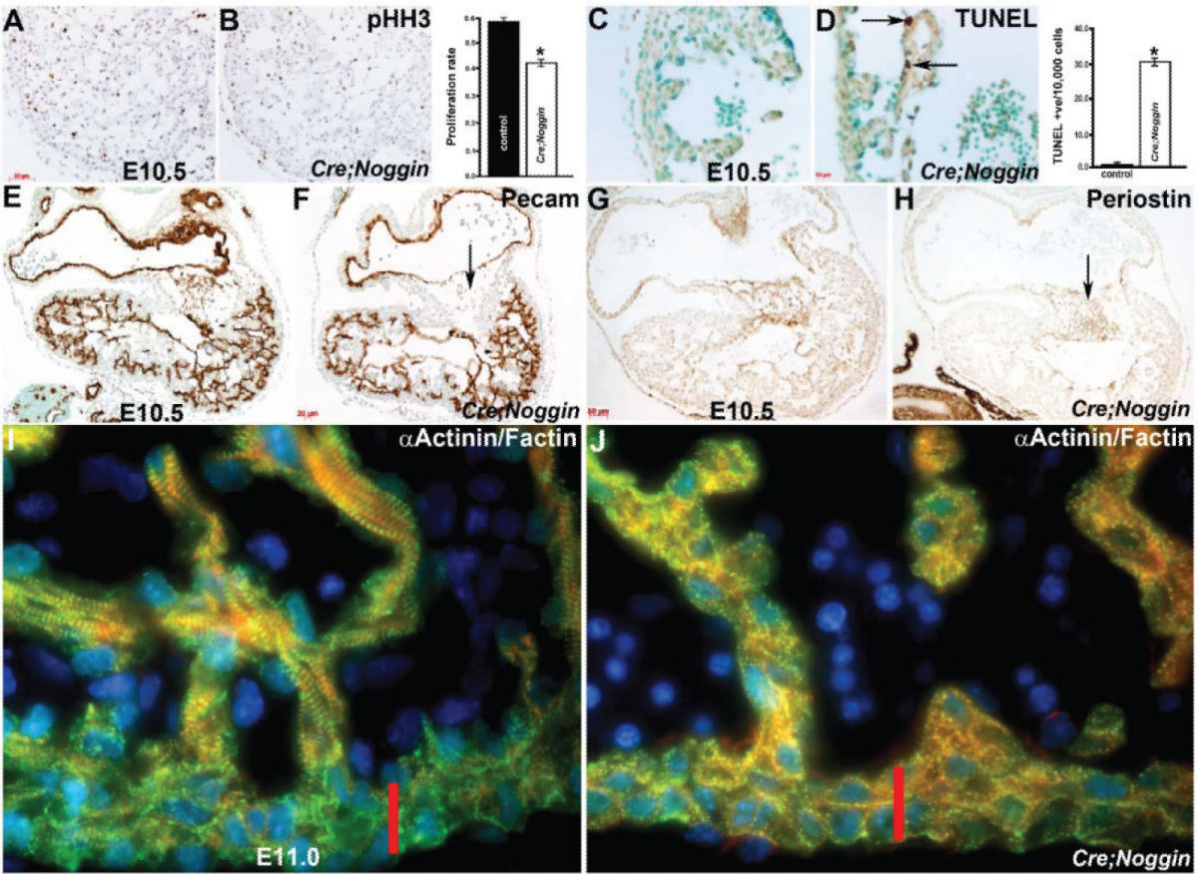
Immunohistochemical analysis of *Nog^End^* mutant growth and development. (**A**,**B**): Phospho-histone H3 examination of cell proliferation revealed that cell proliferation is slightly reduced ~30% within E10.5 mutant hearts, as illustrated via reduced DAB-positive (brown) nuclear staining in mutant (**B**) when compared to control littermate (**A**). Quantitative analysis indicates that the proliferation rate in mutant hearts is significantly reduced compared with the control sample. Data were averaged from 3 independent embryos with error bars indicating SD. * *p* < 0.05 (Student t test); (**C**,**D**): TUNEL analysis revealed that E10.5 mutant (**D**) hearts exhibit apoptotic cells (arrows in (**D)**) within the trabeculae adjacent to the overlying *lacZ*-postive endocardium compared to zero apoptosis in control hearts (**C**); Quantitative analysis indicates that apoptosis is only observed in the mutant ventricle. Data were averaged from 3 independent embryos with error bars indicating SD. * *p* < 0.05 (Student t test); (**E**,**F**): Expression of the endothelial cell adhesion and signaling receptor PECAM-1 confirms that the endocardium is still present and intact within *Nog^End^* heart atria and ventricles, and that the mutant endocardial cells overlying the hypoplastic cushions (arrow in (**F**)) is unaffected. Note that the weaker PECAM-1 immunoreactivity in the control cushion is non-specific background staining, as endocardial cells undergoing EMT exhibit minimal to no pECAM-1 staining; (**G**,**H**): Reduced expression of the TGFβ -responsive endocardial cushion marker Periostin indicates that mutant endocardially-derived cushions are anomalous (arrow in (**H**)) compared to control littermates (**G**); (**I**,**J**): Confocal immunofluorescent imaging of F-actin (red), Actinin (green) with DAPI nuclear staining (blue), reveals that while these cytoskeletal proteins are present within both control (**I**) and mutant (**J**) hearts, there are far fewer mature contractile fibrils within the E11 *Nog^End^* mutant cardiomyocytes when compared to littermate controls. Moreover, the normal stratification of mature contractile apparatus within primarily the trabeculae rather than compact zone cardiomyocytes (indicated via red bar) observed in control heart (**I**) is not present in *Nog^End^* mutant hearts (**J**); Scale bars: (**A**,**B**) = 50 μm; (**C**,**D**) = 10 μm; (**E**–**H**) = 20 μm.

As *Nog^End^* mutant hearts are smaller than control littermates, we sought to determine if the reduction in *Nog^End^* heart size was a result of decreased proliferation, increased apoptosis, or both we used phospho-histone H3 (pHH3) immunostaining and TUNEL analysis (Figure 3). Significantly, proliferation within *Nog^End^* mutant hearts was reduced ~30% (*Nog^End^* 3005 ± 8 pHH3/9134 ± 17 total cells *vs.* control 4454 ± 13 pHH3/9473 ± 25 total cells; *p* 0.017) at E10.5 (Figure 3C,D), following counting of DAB-positive nuclear labelling compared to total nuclei counted from twelve sections of individual samples of wildtype controls and mutants (*n* = 3 for each genotype and 6 slides/embryo). Moreover, TUNEL analysis revealed that E10.5 mutant hearts exhibit apoptotic cells within the trabeculae adjacent to the overlying endocardium compared to zero apoptosis in control hearts (Figure 3D). Combined, the pHH3 and TUNEL data suggest that ectopic *Noggin* within the endocardium principally results in a smaller *Nog^End^* heart size from E10.5 onwards due to a reduced cell proliferation rate, as well as subsequent programmed cell death in isolated *Nog^End^* cardiomyocytes. As *Nfatc1^Cre^* (Figure 2) nor ectopic *Noggin* (Figure 1) are present within the cardiomyocyte lineage, this suggests that bystander or secondary signaling suppression from endocardium to adjacent cardiomyocytes is disturbed in *Nog^End^* hearts.

In order to examine the integrity of the endocardium and to begin to assess the effects of ectopic *Noggin* upon endocardial gene expression and differentiation, we used immunohistochemistry to evaluate endothelial cell adhesion and signaling receptor PECAM-1 expression (also called CD31). As PECAM-1 is one of the earliest adhesion molecules whose expression is restricted to presumptive endothelial and endocardial cells [49], unaffected PECAM-1 staining confirms that the endocardium is still present and intact within *Nog^End^* heart atria and ventricles, and that the mutant endocardial cells overlying the hypoplastic cushions is unaffected (Figure 3F). Confirming abnormal endocardial cushion morphogenesis and reduced phosphoSMAD3 suppression, *Nog^End^* hearts exhibit reduced TGFβ -responsive endocardial cushion marker Periostin (Figure 3H).

To understand the basis for the *Nog^End^* bradycardia and cardiac failure leading to *in utero* lethality, we analyzed myofibril genes important for early cardiac contraction and generation of the heartbeat. As Smooth muscle actin is one of the first and transiently expressed microfilament proteins in the embryonic heart [50] we examined its expression first. Significantly, SMA was unaltered in *Nog^End^* hearts and was not ectopically expressed (not shown). Subsequently, we used confocal analysis to examine F-actin (linear filament expression) and Actinin (localized to the Z-disk) expression within control and mutant cardiomyocyte contractile apparatus in detail. Although both these cytoskeletal proteins were present in E10 and E11 control and mutant cardiomyocytes, there are noticeably fewer mature contractile fibrils within the E11 *Nog^End^* mutant cardiomyocytes when compared to littermate controls (Figure 3I,J). Moreover, the normal stratification of mature contractile apparatus within primarily the trabeculae rather than compact zone cardiomyocytes seen in control hearts, was not observed in *Nog^End^* mutant hearts (Figure 3J). These data suggest that *Nog^End^* myofibrillar maturation and expansion of the contractile apparatus may be compromised via ectopic *Noggin* expression in adjacent endocardium and suppression of TGFβ superfamily signaling resulting in contractile dysfunction.

### 3.5. Nfatc1^Cre^ Lineage is Required for Heart Development and in Utero Survival

**Figure 4:**
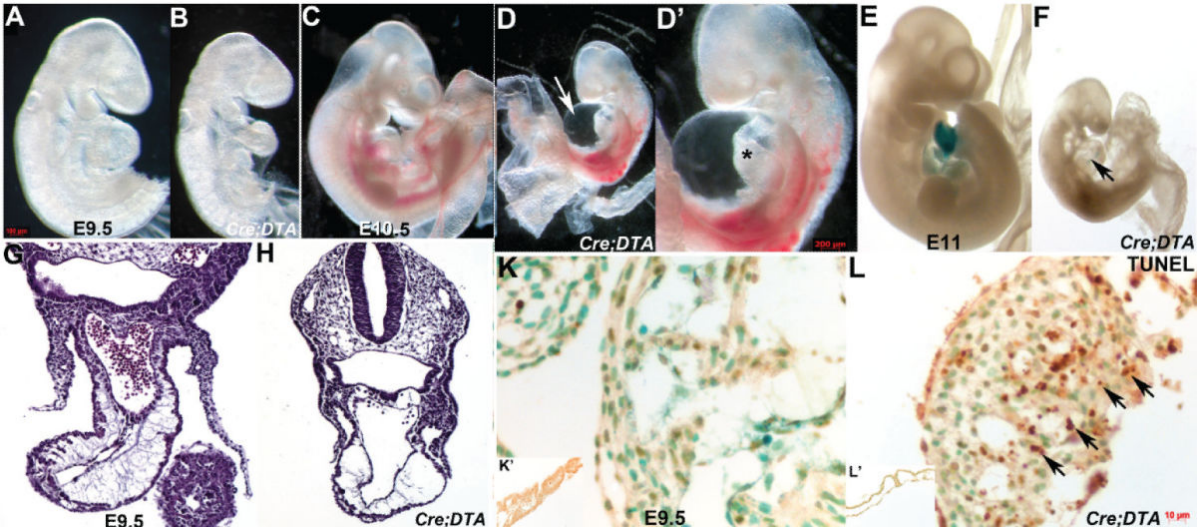

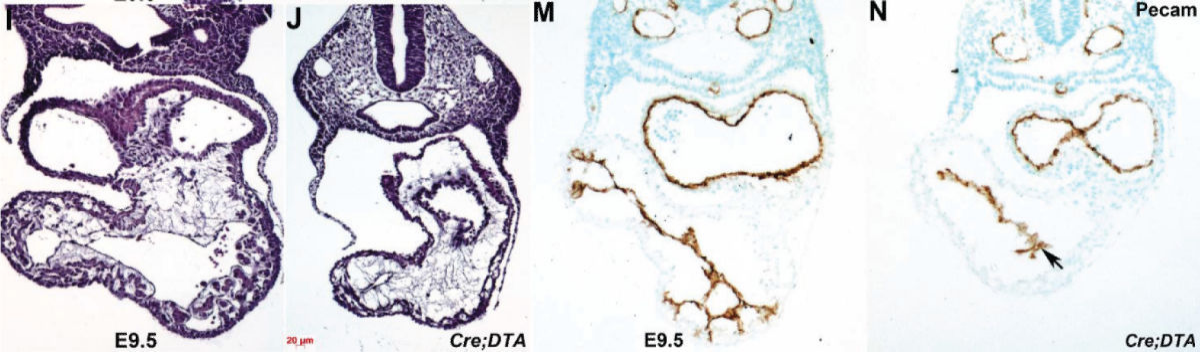
*Nfatc1^Cre^* lineage is required for heart development and *in utero* survival. (**A**–**D**): Whole embryo analysis following genetic cell ablation mediated via *Nfatc1^Cre^*;*R26r^DTA^* results in a smaller heart by E9.5 (**B**) and severe runting, pericardial effusion (indicated via arrow in **D**), widespread hemorrhaging and pooling of blood at E10.5 (**D**,**D’**) when compared to control littermates. Growth retardation of the pharyngeal arches and heart is detectable by E9.5 and by E10.5 the ablation mutants are dying and have very small dysplastic linear hearts (**D’**) compared with controls (**C**); (**E**,**F**): Lineage mapping at E11 in control *Nfatc1^Cre^*;*R26r* and ablated *Nfatc1^Cre^*;*R26r^DTA^;R26r* mutants confirms that the vast majority of the *Nfatc1^Cre^* marked endocardial lineage is absent (no *lacZ* in heart, arrow in (**F**)) and that *Cre* expression is absent from the control yolk sac (**E**); (**G**–**J**): Histology reveals that ablated linear hearts (**H**,**J**) are smaller and abnormal and that there is severe reduction of cardiomyocytes and very little trabeculae compared to control littermates (**G**,**I**) in transverse H&E stained sections through the E9.5 outflow tract (**G**,**H**) and atrioventricular canal planes (**I**,**J**); (**K**,**L**): TUNEL analysis revealed that E9.5 mutant (**L**) hearts exhibit extensive apoptosis in both endocardial cells and cardiomyocytes (arrows in (**L**)) in ablated heart compared to no apoptosis in control (**K**); **K’** and **L’** inserts are low power views of the control (**K’**) and ablated mutant (**L’**) yolk sacs. Note there is no apoptosis within either yolk sac but that the ablated mutant yolk sac does not have mature vessels; (**M**,**N**): Expression of the endocardial marker PECAM-1 confirms that the endocardium is still present and intact in both E9.5 control (**M**) and ablated mutant (**N**) atria and ventricles. Scale bars: (**G**–**J**) = 20 μm; (**K**,**L**) = 10 μm.

In order to determine the initial role of the endocardium within the whole embryo and specifically its functional requirement within the complex environment of the developing heart, we used genetic ablation via *Cre/loxP* and lineage-restricted induction of *diphtheria toxin fragment-A* expression. *Nfatc1^Cre^* knockin line was crossed with the *R26r^DTA^* knockin line [35], and the resultant embryos analyzed from E9 onwards (as *Nfatc1^Cre^* expression initiates E9 and DTA-mediated programmed cell death typically takes ~16–20 h for complete cell removal; [35]. Whole embryo analysis following genetic cell ablation revealed that E9.5 *Nfatc1^Cre/DTA^* mutants are runted, have smaller hearts with an absence of a defined right ventricle when comparted to control littermates (Figure 4B) and appeared pale due to a decrease in circulating blood. Furthermore, E10.5 *Nfatc1^Cre/DTA^* mutants are severely growth retarded, exhibit widespread hemorrhaging and pooling of blood. Additionally, all the ablated mutants died between E10.5 and E11 with severe pericardial effusion, suggesting insufficient heart function (Figure 4D). The E10.5 *Nfatc1^Cre/DTA^* mutant hearts were hypoplastic and had failed to loop, resulting in dysmorphic linear hearts. Histology confirmed an absence of normal rightwards looping in ablated hearts, and also revealed that there is severe reduction of cardiomyocytes and almost a complete absence of trabeculae compared to control littermates (Figure 4H,J). As expected, there is significant apoptosis present in *Nfatc1^Cre/DTA^* hearts, particularly within the trabeculae forming region (Figure 4L). Significantly, although lineage mapping at E11 in *Nfatc1^Cre^*;*R26r^DTA^;R26r* mutants confirms that the vast majority of the *Nfatc1^Cre^* marked endocardial lineage is absent, PECAM-1 staining confirms that there is still an endocardium present and mostly intact in E9.5 ablated mutant atria and ventricles (Figure 4N). Combined, these results substantiate that *Nfatc1^Cre^* lineage endocardial progenitors are essential for *in utero* survival and principally required during the trabeculation phase and endocardial cushion formation during cardiac development, likely through its functional regulation and support of adjacent cardiomyocytes.

### 3.6. qPCR mRNA Profiling and in Situ Verification of Expression Alterations

**Figure 5:**
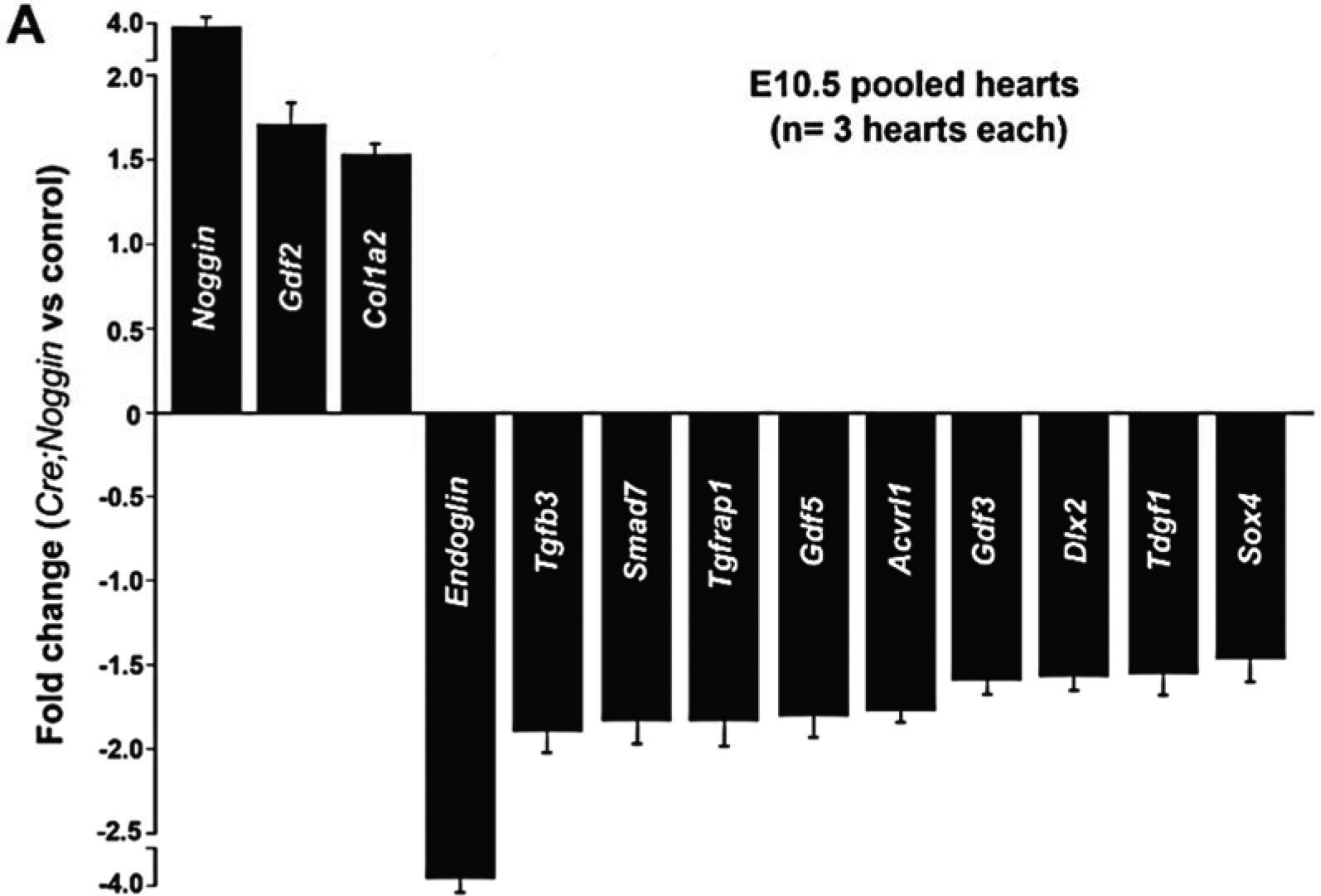

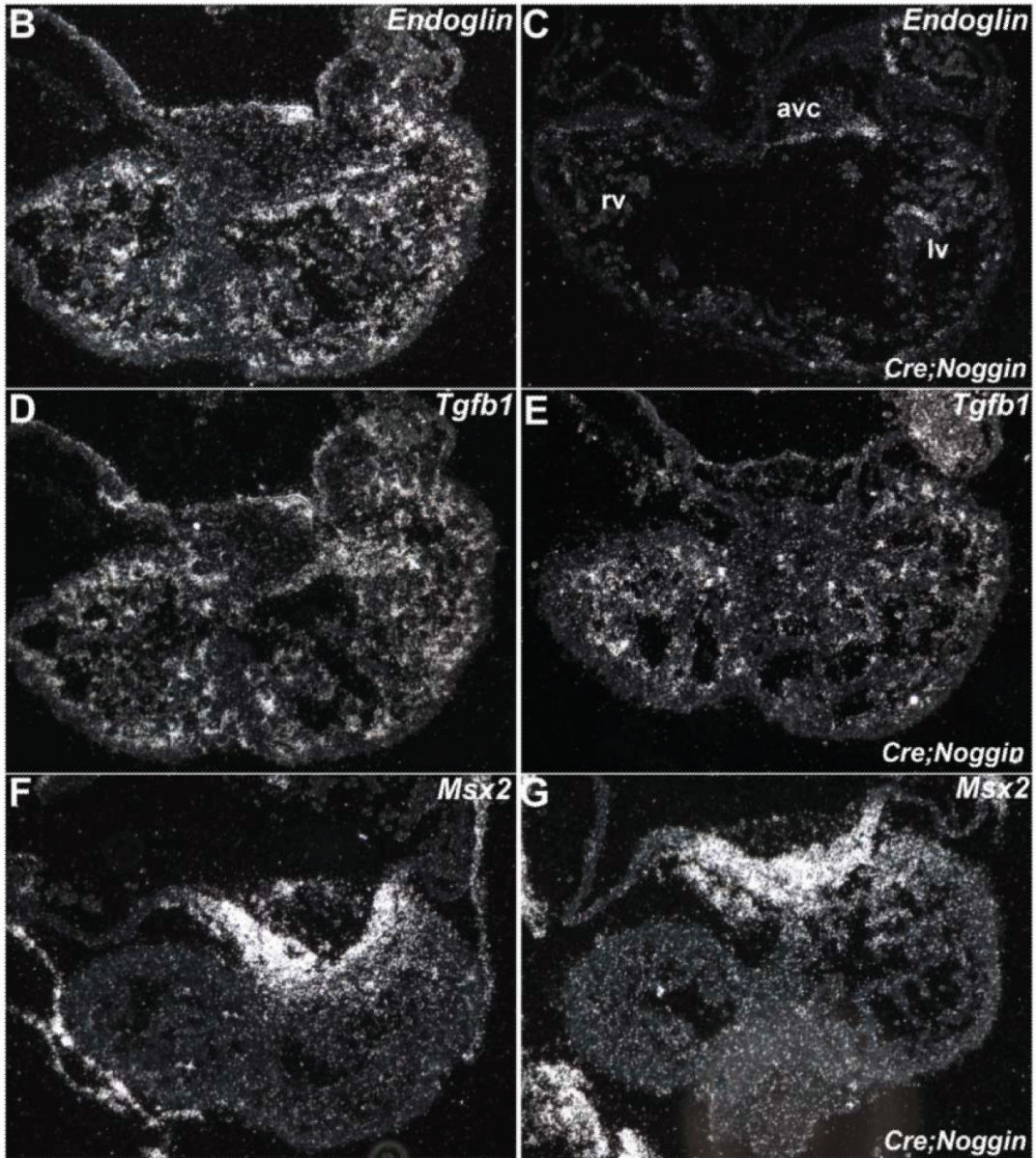
qPCR mRNA profiling and *in situ* verification of expression alterations. (**A**): Quantitative PCR using a custom array of 84 genes responsive to TGFß superfamily signal transduction revealed statistically significant changes in transcript levels of the 13 listed genes. Data are presented as a logarithmic plot of relative expression (mutant/wildtype), where a value of 1 indicates no difference between E10.5 *Nog^End^* mutant and wildtype pooled hearts and values <1 indicate reduced expression. The biggest alterations were observed in *Noggin* levels, which were x4 fold elevated in mutant hearts; and *Endoglin* levels, which were×3.7 fold reduced in mutant hearts. Error bars represent SEM; (**B**,**C**): Reduced *Endoglin* expression levels were verified by radioactive *in situ* hybridization, as the E10.5 mutant heart (**B**) exhibits significantly less *Endoglin* mRNA throughout the endocardial lineage when compared to wildtype littermate (**B**); (**D**–**G**): As qPCR profiling did not reveal any alterations in either endocardially-restricted *Tgfβ1* or myocardial *Msx2* expression levels, we used *in situ* hybridization to examine whether spatial alterations were present in mutant hearts; Radioactive *in situ* hybridization revealed comparable *Tgfβ1* mRNA expression in control (**D**) and mutants (**E**), and *Msx2* was normally expressed within the controls (**F**) and *Nog^End^* mutant (**F**) myocardium adjacent to the AV cushions. Abbreviations: avc, atrioventricular cushion; lv, left ventricle; rv, right ventricle.

To identify the endocardial targets that are altered via ectopic *Noggin* and the factors that may mediate endocardial-cardiomyocyte signaling during cardiac morphogenesis, we examined expression of TGFβ superfamily targets and genes known to be involved in BMP and TGFβ ligand downstream signaling by qPCR in isolated E10.5 hearts (*n* = 3 pooled isolated *Nog^End^* and wildtype hearts, carried out in duplicate samples). The E10.5 stage was chosen as *Nog^End^* mutants do not exhibit an overt phenotype at this age (Figure 1D), but do show pSMAD deviations (Figure 1K). Quantitative PCR using a custom array of 84 genes related to TGFβ /BMP-mediated signal transduction, including SMAD, SMAD target genes, adhesion and extracellular molecules and transcription factors revealed significant changes in transcript levels of the 13 listed genes (Figure 5A). As proof of principle, it was significant that expression of *Noggin* was elevated almost×4 fold in *Nog^End^* hearts. Meaningfully, neither *Bmp2* nor *Bmp4* were affected, and endocardially-restricted *Tgfβ1,* myocardial *Tgfβ2* and *Msx2* levels were unaltered. Similarly, *Smad1-5* expression levels were normal. However, *Tgfβ3* mRNA was downregulated ×1.9 fold in *Nog^End^* hearts (Figure 5A).

This analysis further demonstrated that expression of Growth differentiation factor 2 (*Gdf2*), which is also known as embryonic liver-specific endothelial *Bmp9*, is upregulated ×1.7 fold in *Nog^End^* hearts suggestive of misspecification. Collagen alpha-2(I) chain (*Col1a2*) expression is also upregulated ×1.5 fold in *Noggin* overexpressing hearts, signifying excessive fibrillar collagen in *Nog^End^* hearts. Most significant was the ×3.7 fold downregulation of endocardially-expressed *Endoglin*, a cell surface glycoprotein within the TGFβ receptor complex [51], within *Nog^End^* hearts compared to control hearts (Figure 5A,C). The downregulation of *Endoglin* within the *Nog^End^* endocardium was verified via *in situ* hybridization (Figure 5C). Correspondingly, *Endoglin*-null embryos with heart valve and septation defects do not progress beyond E10.5, fail to form mature blood vessels in the yolk sac, lack trabeculae and exhibit small hearts with an absence of a defined right ventricle and pericardial effusion [52]. Thus, systemic loss of *Endoglin* in mice phenocopies the *Nog^End^* and *Nfatc1^Cre^*; *R26r^DTA^* embryonic phenotypes and are similarly lethal prior to the fetal stage. Moreover, Activin receptor-like kinase-1/ALK1 (*Acvrl1*) is downregulated ×1.8 fold, and both *Acvrl1* and *Endoglin* mutations are associated with Hereditary Haemorrhagic Telangiectasia diseases, characterized by bleeding from vascular malformations [52–54]. In contrast to *Gdf2* upregulation, both widely expressed BMP-responsive *Gdf3* and *Gdf5* are downregulated. Similarly, BMP-responsive *Sox4* and *Dlx2* developmental process genes are downregulated in *Nog^End^* hearts. Both TGFβ superfamily co-receptors *Tdgf1* (also called *Cripto-1*) and TGFβ 1-receptor Associate Protein-1 (*Tgfrap1*) are downregulated via ectopic *Noggin* expression within the mutant heart endocardium. Finally, the pan-TGFβ superfamily signaling negative regulator *Smad7* [45,55] is also downregulated×1.85 fold in *Nog^End^* hearts. Thus overall, expression profiling indicated that ectopic *Noggin* within the endocardium elicited a specific expression profile response in *Nog^End^* hearts, rather than wholesale suppression of TGFβ /BMP-mediated signal transduction. Moreover, despite Noggin being known as principally a secreted extracellular BMP antagonist [46,47], both BMP and TGFβ downstream effectors were altered.

## 4. Discussion

The goal of this study was to determine the functional effects of loss of the endocardial lineage and ectopic Noggin-mediated suppression of BMP signaling during initial heart morphogenesis, trabeculation and endocardial cushion formation. Although endogenous Noggin is transiently expressed in the normal E8.75-9 mouse heart, and is thought to establish conditions conducive to cardiogenesis [19], the consequences of deregulated Noggin within the endocardial lineage are unclear. We utilized two complementary mouse model systems to demonstrate the cell-autonomous requirement of the endocardial lineage and function of unaltered BMP levels in facilitating endothelium-cardiomyocyte cross-talk and promoting mesenchyme formation in endocardial cushions of mouse embryos.

Binary *pMes-Noggin/Nfatc1^Cre^* transgenic mutants exhibit smaller hearts that are bradycardic and all die mid-gestation. *Nog^End^* lethality and slow heartbeat are most likely due to fewer contractile elements, a lack of maturation of the actin-myosin microfilaments and reduced proliferation, resulting in insufficient cardiac output to drive bloodflow throughout the embryo. Although the endocardium has recently been shown to serve as a *de novo* source for transient definitive haematopoietic progenitors [56], the pale appearance of the *Nog^End^* E10.5 mutants is more likely due to a decrease in circulating blood rather than defects in yolk sac vasculogenesis and/or primitive hematopoiesis, especially as *Nfatc1^Cre^*-mediated Cre expression is restricted to the heart [3]. Notably, the Noggin-mediated alteration of contractile apparatus morphogenesis is consistent with *in vitro* studies using cultured chick precardiac mesoendoderm and exogenous Noggin [57], in which SMA expression was unaltered but sarcomeric actinin, titin, sarcomeric myosin were all misexpressed. The hypoplastic *Nog^End^* outflow tract and AVC endocardial cushion phenotype is consistent with *in vitro* data showing that Noggin blocks EMT [18]. Although *Bmp4* null hearts exhibit similar proliferation and growth abnormalities and dysmorphic hearts [58], *cTnT-Cre*-mediated conditional deletion of *Bmp4* does not affect heart size until fetal stages [59]. Similarly, *Nkx2.5^Cre^* conditional deletion demonstrated Bmp2 is required for formation of cardiac jelly and formation of the cardiac cushions [60]. Moreover, *Flk1-Cre* mediated loss of *Alk3 (Bmpr1a)* resulted in embryonic lethality by E11.5, severe abdominal hemorrhaging and endocardial cushion defects [61]. To complement the Noggin transgenic analysis, we further examined the functional requirement of the E9-10 endocardium using *Cre/loxP*-mediated genetic cell ablation via diphtheria toxin-A. Significantly, E9.5 *Nfatc1^Cre/DTA^* mutants are severely runted, exhibit dysplastic linear hearts that hardly beat, pooling of blood and 100% die by E11. While we cannot exclude the possibility that poor function may play a potential role within the looping abnormality, our previous data in which the sodium-calcium exchanger *Ncx1* is deleted and results in an absent heartbeat and lethality at E10, the *Ncx1* null hearts still undergo looping despite lack of any cardiac function [37]. Combined, these results demonstrate Noggin-mediated suppression of BMP signaling within the endocardium, as well as endocardially-restricted diphtheria toxin-A genetic ablation, both have profound affects upon the adjacent myocardium.

As Noggin is usually known as a secreted extracellular BMP antagonist [46,47], it was somewhat surprising that the adjacent trabeculated myocardium and atrioventricular/outflow myocardium was unaffected (Figure 1). This result may be due, in part, to the observation that Noggin is directly expressed within the mutant endocardium and that it becomes detached from the underlying mutant trabeculae (Figure 2) resulting in a reduced ability to secondarily suppress myocardial phosphoSMAD levels, or that the levels of secreted Noggin are too low to suppress the robust phosphoSMAD signaling routinely observed in embryonic cardiomyocytes. In fact Noggin has been shown to exhibit concentration-dependent effects, including inducing downstream targets at low concentrations and repressing those same targets at high concentrations [62,63]. Thus, it is unlikely that the *Nog^End^* myocardial phenotype is just a consequence of excess secreted Noggin secondarily affecting the adjacent myocardium. Rather, our data supports the hypothesis that endocardial-myocardial cross talk may regulate myofibril assembly. Precisely how this achieved and what role BMP signaling plays is presently unclear, but it has been demonstrated the endocardium can act as an active transendothelial physicochemical gradient for various ions [64] and that contractility of isolated cardiac muscle can be profoundly altered via the presence of intact endocardium [65]. Within the embryonic heart there is a close relationship between newly assembling myofibrils and the sarcolemma, and that interactions between the membrane and the myofibril promote the early steps of striated myofibril assembly in both heart and skeletal muscle [66,67]. Perhaps secreted Noggin or reduced endocardial BMP signaling (and consequent Endoglin/ALK1 suppression) may aberrantly affected adjacent myocardial sarcolemma, resulting in lack of maturation of the contractile apparatus. This *in vivo* Noggin-mediated alteration of contractile apparatus morphogenesis is consistent with *in vitro* [57], in which SMA expression was unaltered via exogenous Noggin but sarcomeric actinin, titin, and sarcomeric myosin expression was affected.

Perhaps one of the most interesting observations in this study is that ectopic endocardial Noggin results in suppression of both phosphoSMAD1/5/8 and phosphoSMAD3. As expected, Noggin overexpression resulted in reduced phosphoSMAD1/5/8 levels, confirming suppression of BMP signaling. However, phosphoSMAD3 levels were also suppressed in *Nog^End^* hearts (but not phosphoSMAD2), suggesting parallel suppression of TGFβ signaling. Although we do not yet know whether loss of BMP signaling results in a secondary loss of TGFβ signaling or whether Noggin can directly affect TGFβ signaling within the endocardium and/or adjacent cardiomyocytes, these data clearly demonstrate that phosphoSMAD2 and phosphoSMAD3 can be differentially regulated and phosphoSMAD3 may have a more important developmental role than phosphoSMAD2 in TGFβ superfamily-mediated endothelium-cardiomyocyte cross-talk morphogenesis. These data may also indicate that blocking BMP signaling can disrupt other signaling pathways, thus normal endocardial-cardiomyocyte cross-talk development requires a balance of BMP-TGFβ signaling for normal cardiac morphogenesis. Although Noggin is usually known as a secreted extracellular BMP antagonist [46,47], there are reports that TGFβ inducing the phosphorylation of SMAD1 [68,69], which may reflect redundancy or cooperativity in signaling. Moreover, functional redundancies among endocardially expressed type-I BMP receptors have been suggested by the studies in which *ALK2* or *ALK3* were conditionally deleted [60,61,70,71], as ALK2 can bind TGFβ and Activin ligands in addition to BMPs. Similarly, *Tgfβ2* has been shown to be an endocardial activation marker [72], *Tgfβ2* is downregulated in *Bmp2/Nkx2.5^Cre^* conditional knockout hearts [60] and BMP2 can induce *Tgfβ2* expression within mouse AVC explant assays [18]. Although BMP signaling in the myocardium can also regulate myocardial *Tgfβ2* expression as evident in the *Alk3* conditional knockout [73], it is clear that endothelium-cardiomyocyte cross-talk regulation involves the TGFβ superfamily as a whole. Indeed, *Tgfβ2* knock-out mice, but not *Tgfβ1* or *Tgfβ3* knock-out mice, feature deficient EMT in the cardiac cushions initially [74] but subsequently exhibit enlarged hyperplastic cushions/valves. Significantly, *Tie1-Cre* endocardial-restricted loss of *Smad4* results in an absence of EMT, acellular cushions, and reduced endocardial proliferation [23]; as well as severe runting, blood pooling in E11.5 mutants and 100% lethality by E12.5. Given that SMAD4 is common to both TGFβ and BMP signaling, this further supports the data that both TGFβ and BMP signaling play a role within the endocardium during early heart formation and unperturbed cross-talk is required for *in utero* survival.

To extend the *in vivo* genetic analysis and phenotyping of mutant hearts, we examined the resultant TGFβ -BMP-mediated signal transduction expression profiles elicited via ectopic Noggin expression within the endocardial lineage. In addition to *Noggin* upregulation and mis-expression of eleven other effects (Figure 5), this work clearly shows that expression of Endoglin is robustly repressed within the endocardium. As systemic *Endoglin* null embryos phenocopy the *Nog^End^* mutants and die at E11 with enlarged thin-walled ventricles, dilated OFT, cardiac cushions that lack mesenchyme cells, and angiogenic defects in yolk sac [52], Endoglin is a potential, if not likely, candidate for mediating the endocardial-cardiomyocyte cross-talk defects observed. The non-signaling receptor Endoglin is thought to function as a regulator of the cell-extracellular microenvironment, specifically within the process of EMT and TGFβ superfamily regulated activation and invasion pathways during cardiac development [75]. Indeed, co-expression of Endoglin is thought to influence which TGFβ isoforms are bound and which signaling responses are made [76], and in addition to TGFβ s, Endoglin can also interact with Activin-A, BMP7 and BMP2 ligands. Moreover, our expression profiling also revealed that *Acvrl1 (ALK1)* type I TGFβ receptor, which appears to signal through SMAD1 [77], is similarly downregulated. Interestingly, both ALK1 and Endoglin exhibit similar endothelial expression patterns and both act within the same signaling pathway resulting in hereditary hemorrhagic telangiectasia disease [78,79] and arteriovenous malformations [80]. While we do not know how endocardial BMP suppression directly regulates expression of Endoglin in the *Nog^End^* endocardium or whether this is a primary consequence of reduced phosphoSMAD levels, these studies indicate that both BMP and TGFβ downstream effectors were altered. Similarly, how endocardial BMP-TGFβ superfamily-mediated *Endoglin* suppression alters adjacent myocardial expression profiles still needs to be investigated.

## 5. Conclusions

Together with previous studies [23,24,74], our study suggests reciprocal BMP-TGFβ signaling between the endocardium and myocardium during early heart morphogenesis is critical for cardiac development, specifically seeding of the endocardial cushions, trabeculation and maturation of the contractile apparatus to enable maintenance of cardiac output and *in utero* survival. Expression profiling of these ectopic Noggin expressing *Nog^End^* mutant hearts reveals that this endocardial-myocardial signaling involves coordinated BMP-TGFβ superfamily-mediated maintenance of Endoglin/ALK1 endocardial expression and consequent regulation of myocardial growth and promotion of endocardial EMT to enable population of the cardiac cushions. Combined, these results demonstrate that fine-tuning of TGFβ superfamily signaling within the endocardium is required for essential cellular and paracrine contributions to the growth of the embryonic myocardium and the development of the cardiac valves.
